# Lack of evidence of the interaction of the Aβ peptide with the Wnt signaling cascade in *Drosophila* models of Alzheimer’s disease

**DOI:** 10.1186/s13041-014-0081-y

**Published:** 2014-11-12

**Authors:** Anne-Marie Lüchtenborg, Vladimir L Katanaev

**Affiliations:** Department of Pharmacology and Toxicology, Faculty of Biology and Medicine, University of Lausanne, Rue du Bugnon 27, Lausanne, 1005 Switzerland

**Keywords:** Alzheimer’s disease, Aβ peptide, *Drosophila*, Wnt signaling

## Abstract

**Background:**

Alzheimer’s disease (AD) is the leading form of dementia worldwide. The Aβ-peptide is believed to be the major pathogenic compound of the disease. Since several years it is hypothesized that Aβ impacts the Wnt signaling cascade and therefore activation of this signaling pathway is proposed to rescue the neurotoxic effect of Aβ.

**Findings:**

Expression of the human Aβ42 in the *Drosophila* nervous system leads to a drastically shortened life span. We found that the action of Aβ42 specifically in the glutamatergic motoneurons is responsible for the reduced survival. However, we find that the morphology of the glutamatergic larval neuromuscular junctions, which are widely used as the model for mammalian central nervous system synapses, is not affected by Aβ42 expression. We furthermore demonstrate that genetic activation of the Wnt signal transduction pathway in the nervous system is not able to rescue the shortened life span or a rough eye phenotype in *Drosophila*.

**Conclusions:**

Our data confirm that the life span is a useful readout of Aβ42 induced neurotoxicity in *Drosophila*; the neuromuscular junction seems however not to be an appropriate model to study AD in flies. Additionally, our results challenge the hypothesis that Wnt signaling might be implicated in Aβ42 toxicity and might serve as a drug target against AD.

## Findings

Alzheimer’s disease (AD) is a major neurodegenerative malady, affecting today more than 35 million people worldwide with the tendency to double in the prevalence every twenty years [1]. The two major hallmarks of AD are the intracellular neurofibrillary tangles consisting of the hyperphosphorylated tau protein and the extracellular plaques mainly containing the aggregated Aβ peptide. According to the amyloid hypothesis, Aβ peptides in their various aggregation states are the major pathogenic compounds in AD.

Already more than a decade ago, first experiments suggested the interaction of Aβ with the Wnt (Wingless [Wg] in *Drosophila*) signaling cascade and its contribution to Aβ toxicity [2,3]. Wnt signaling is involved in numerous developmental processes and regulates synaptic formation and stability in the adult organism [4]. In the canonical pathway, the ligand Wnt activates the receptor Frizzled (Fz) and its co-receptor LRP5/6 to induce reorganization of the β-catenin-destruction complex, a protein complex consisting of Axin, APC, glycogen synthase kinase 3β (GSK3β, Shaggy [Sgg] in *Drosophila*) and casein kinase, through the scaffolding protein Dishevelled and the trimeric Go protein [4,5]. Thus Wnt signaling leads to stabilization of β-catenin and its translocation to the nucleus where it induces transcription of Wnt target genes. GSK3β also phosphorylates tau and might be the integration point of Aβ and tau induced toxicity [6].

Lithium is a well-established drug against psychiatric disorders that inhibits, amongst other targets, GSK3β [7]. Due to its neuroprotective effect, it has been used in small-scale trials in patients with AD, although with contradictory results [8]. In transgenic mouse models of AD, lithium treatment reduced behavioral impairments and the Aβ load in mouse brains [9]. Likewise, the destabilization of cytosolic β-catenin and the neurotoxicity induced by Aβ in cell culture could be attenuated by LiCl, potentially implying Wnt signaling in Aβ toxicity [10]. Additionally, incubation with Wnt3a reduces the neurotoxic effect of Aβ in cell culture assays – an effect mediated by Fz1 [11,12]. Furthermore, it has been demonstrated that the Aβ peptide can bind to the receptor Fz5 [13]. Therefore, the Wnt cascade was suggested to serve as a potential drug target against AD [3].

Most investigations on Wnt signaling and Aβ have mainly been carried out in mice and cell culture where the inhibition or activation of the signal pathway can be achieved pharmacologically. The genetic model organism *Drosophila melanogaster* has also been used to study the mechanisms of AD [14]. In this model, several possibilities to mimic AD are available; amongst others the neuronal expression of human Aβ42 peptide [15,16]. These flies recapitulate several aspects of AD observed in patients: they show learning deficits, reduced locomotion, shorter life span and neurodegeneration and amyloid deposition in the brain [15].

However, the link between the Wnt signaling and Aβ has not been so far investigated using the *Drosophila* models. We have recently provided an in-depth characterization of the Wg-Fz2-Go-Ankyrin2 signaling pathway active on the presynaptic side of *Drosophila* neuromuscular junctions (NMJs) [17]. NMJs are composed of synaptic boutons – circular structures containing active zones for neurotransmitter release. Being a glutamatergic synapse (unlike most other synapses in *Drosophila*), NMJ provides an especially useful model for mammalian synapses [18]. Expression of human Aβ42 in *Drosophila* NMJs has already been performed and reports to induce defective NMJ formation and functioning [19–21]. Thus we aimed at investigation of the details of the expected interaction of Aβ42 and the Wnt signaling pathway in this system.

**Figure 1:**
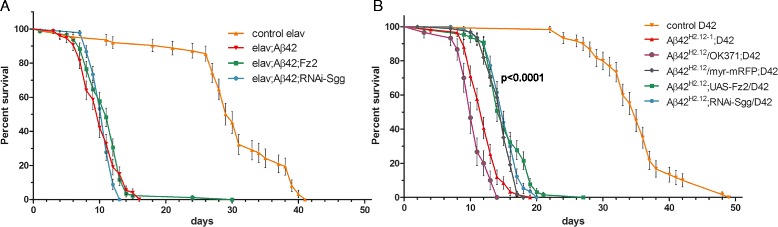
**Expression of Aβ42 in the nervous system dramatically reduces the life span. (A-B)** Survival curves of the indicated genotypes expressed either with the pan-neuronal driver *elav-Gal4* **(A)** or the driver *D42-Gal4* which expresses in the glutamatergic motoneurons **(B)**. P < 0.0001 comparing the controls *elav-Gal4* and *D42-Gal4* to Aβ42 expression, respectively. p = 0.0028 comparing *Aβ42; D42-Gal4* and *Aβ42/OK371-Gal4; D42-Gal4*. Co-expression of Fz2 or RNAi-Sgg did not rescue life span. P-values are calculated with the Log-rank test.

Pan-neuronal expression of secreted Aβ42 (using the *elav-Gal4* driver) has previously been shown to reduce the life span of *Drosophila* [15,16]. We recapitulated these findings (Figure 1A) and further showed that a similar reduction in the life span can be achieved through Aβ42 expression by a motoneuron-specific driver *D42-Gal4* (Figure 1B). Indeed, Aβ42 caused a reduction of the median survival from 30 days (*elav-Gal4* control, n = 62) to 10 days (*elav-Gal4; UAS-Aβ42*, n = 74) (p < 0.0001, Log-rank test) and of the maximal life span from 42 to 16 days when the pan-neuronal driver was used (Figure 1A), and from 35 days (*D42-Gal4* control, n = 60) to 12 days (*D42-Gal4; UAS-Aβ42*, n = 59) (p < 0.0001, Log-rank test) and the maximal life span from 49 to 19 days when the motoneuron-specific driver was used (Figure 1B). This effect is dose-dependent: increasing the amount of Gal4 produced per cell by adding another motoneuron-specific driver *OK371-Gal4* to *D42-Gal4* to express the Aβ42 peptide further reduced the median life span to 10 days (p = 0.0028, Log-rank test) and the maximal survival to 14 days (Figure 1B). Cumulatively, these findings suggest that the major effect of Aβ42 on the life span observed previously [15,16] by the pan-neuronal Aβ42 expression takes place in glutamatergic neurons.

**Figure 2:**
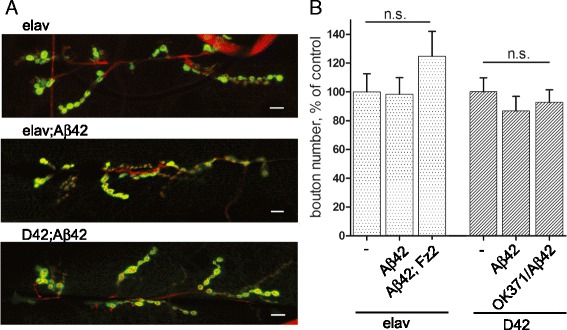
**The morphology and bouton number of NMJ are unaffected by neuronal Aβ42 expression. (A)** Representative images of NMJs on muscle 6/7 of *elav-Gal4*, *Aβ42; elav-Gal4*, and *Aβ42; D42-Gal4* stained with Dlg to visualize the postsynaptic side and HRP to visualize the neuron. Aβ42 expression does not change NMJ morphology. **(B)** quantification of the bouton number as mean ± sem in percent of control. n.s. means ‘not significant’ compared to control as calculated with student’s t-test.

We further investigated this effect of Aβ42 by analyzing the morphology of the NMJs in *Drosophila* larvae. We expressed Aβ42 through *elav-Gal4*, *D42-Gal4*, and the combination of *D42-Gal4* and *OK371-Gal4* to increase the expression levels in the motoneurons. In contrast to previously published results where Aβ42 expression induced small morphological changes in NMJs and expression of human APP and BACE led to a reduction in bouton number [20,21], we could not detect an influence of Aβ42 on the larval synapse. Both in terms of the overall morphology (Figure 2A) and in bouton number (Figure 2B), NMJs appear to be unaffected by Aβ42.

We further aimed at investigating the potential interaction of Aβ42 and the Wnt signaling cascade. Given the absence of the expected phenotypes of Aβ42 expression on NMJ morphology and bouton numbers (Figure 2), we could not use this readout to study the potential interaction between Aβ42 and the pathway. We thus decided to use the life span reduction as the readout. To this end, we co-expressed Aβ42 together with Fz2 – the main Wnt receptor in the *Drosophila* nervous system [17] – or together with the RNAi construct targeting Sgg to activate Wg signaling [17]. We used both the *elav-Gal4* and *D42-Gal4* drivers. We also tried co-expression of Aβ42 with the constitutively active form of Gαo (Gαo [Q205L]) – the Fz2 transducer in the NMJs [17], but this was lethal with either driver, probably due to involvement of the trimeric Go protein in other neuronal activities.

In the motoneurons, co-expression of Fz2 or RNAi-Sgg together with Aβ42 slightly increased the survival compared to Aβ42 expression alone (Figure 1B). However, this was due to a titration effect since an unrelated protein (myr-mRFP) was also able to similarly rescue the life span. In pan-neuronal expression, neither Fz2 nor RNAi-Sgg could significantly increase the median survival, although the maximum life span was increased upon co-expression of Fz2 (Figure 1A). Cumulatively, these results indicate that overactivation of the Wnt signaling transduction pathways in neurons using the genetic tools available in *Drosophila* does not rescue the toxicity (manifested by a shortened life span) induced by secreted Aβ42.

**Figure 3:**
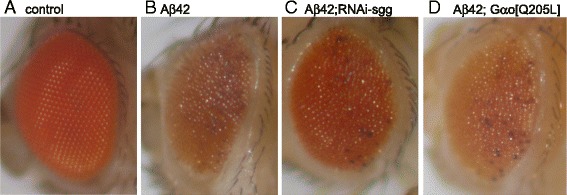
**The rough eye phenotype induced by Aβ42 is not rescued by RNAi-Sgg or Gαo [Q205L].** **(A)** In the control eye (Aβ42 without driver) ommatidia are arranged in a regular array. The other parental line, *GMR-Gal4*, also shows similar wild-type arrangement. **(B)** Expression of Aβ42 in the eyes with the driver *GMR-Gal4* results in a rough eye phenotype. This is not rescued by co-expression of neither RNAi-Sgg **(C)** nor Gαo [Q205L] **(D)**.

This conclusion is further corroborated using another *Drosophila* readout – insect’s eye. Aβ42 expression in the eyes using the *GMR-Gal4* driver leads to a rough eye phenotype (Figure 3, [16]). We co-expressed RNAi-Sgg and Gαo [Q205L] with Aβ42 and could not observe a rescue of the eye roughness (Figure 3). This confirms in a different setting that genetic activation of the Wnt signaling cascade does not rescue Aβ42 induced toxicity in *Drosophila*.

However, it has been previously reported that expression of the dominant-negative form of Sgg, SggS9E, rescues the shortened life span of *Drosophila* that express the arctic Aβ42 peptide [22]. The arctic peptide was shown to decrease Ser9 phosphorylation of Sgg and thereby upregulate its activity [22]. In contrast another study shows no change in phosphorylation of Sgg when wild-type Aβ42 is expressed [20]. The arctic variant accumulates intracellularly in mice [23,24]; therefore it is likely that the arctic variant of Aβ42 exerts a different pathogenic mechanism than the wild-type peptide. Our data suggest that secreted wild-type Aβ42 acts primarily on glutamatergic neurons in *Drosophila*, but induces the toxicity independently from Wnt signaling.

Taken together, our results demonstrate that Aβ42 expression in glutamatergic neurons is responsible for the dramatic shortened life span manifested in *Drosophila* models of AD. However, the glutamatergic NMJs seem not to be appropriate to study the Aβ42-induced changes on a single cell level. In addition, our genetic interaction analysis challenges the widely accepted idea that Aβ42 inhibits Wnt signaling and that Wnt pathway overactivation might reduce the Aβ42 toxicity. This concept is based on numerous studies, mainly relying on the usage of LiCl and its effects on the AD phenotypes. We suggest that caution is taken when interpreting these data, as LiCl is not a specific inhibitor of GSK3β/Sgg, and further since this kinase has many other functions outside the Wnt signaling pathway [7].

## Methods

### Life span, eye analysis and *Drosophila* stocks

For the life span test, flies were crossed at 25°C and 5 male and 5 female newly hatched flies were pooled and transferred to 28.5°C. Flies were transferred to fresh food every other day and time-to-death was recorded for individual flies. 59 to 90 flies were analyzed for each genotype. Analysis of survival was performed with GraphPad Prism 5, p-values were calculated with the Log-rank test.

Eye phenotypes were analyzed after crossing to *GMR-Gal4* at 25°C.

The following stock were used: *elav-Gal4*, *D42-Gal4*, *GMR-Gal4* (all from Bloomington stock center), *UAS-RNAi-Sgg* (VCRC #7005), *UAS-Aβ42* [16]. The lines * OK371-Gal4*, *UAS-Fz2*, and *UAS-Gαo [Q205L]* were used as described [17].

### Immunohistochemistry

For the analysis of the neuromuscular junctions, crosses were set up at 28.5°C and wandering third instar larvae were dissected and stained as previously described [17]. Primary antibodies were: Cy3-coupled goat anti-HRP (123-165-021, Jackson ImmunoResearch) at 1:200 and mouse anti-Dlg (4 F3, Developmental Studies Hybridoma Bank) at 1:100. Boutons were identified based on presynaptic HRP and postsynaptic Dlg staining. Statistical analysis was performed with GraphPad Prism 5. Data are present as mean ± sem.
